# Enhancing attraction of the vector mosquito *Aedes albopictus* by using a novel synthetic odorant blend

**DOI:** 10.1186/s13071-019-3646-x

**Published:** 2019-07-30

**Authors:** Lihua Xie, Wenqiang Yang, Hongmei Liu, Tong Liu, Yugu Xie, Feng Lin, Guofa Zhou, Xiaohong Zhou, Kun Wu, Jinbao Gu, Guiyun Yan, Xiao-Guang Chen

**Affiliations:** 1 Department of Pathogen Biology, Guangdong Provincial Key Laboratory of Tropical Disease Research, School of Public Health, Southern Medical University, Guangzhou, China; 2 Program in Public Health, University of California Irvine, Irvine, California USA

**Keywords:** Olfaction, Host-seeking behavior, Attractive odor blend, Orthogonal design, Mosquito traps, *Aedes albopictus*

## Abstract

**Background:**

The Asian tiger mosquito, *Aedes albopictus*, an increasingly relevant arboviral vector, has spread worldwide. However, currently available tools are limited in terms of effective monitoring of vector populations and accurate determination of the extent of viral transmission, especially before and during outbreaks. Therefore, it is essential to develop novel monitoring and surveillance tools, particularly those that target adult mosquitoes and enhance the trapping efficiency for *Ae. albopictus*.

**Methods:**

A variety of human body odorants associated with different types of mosquito olfactory receptors were selected, and their attractiveness to *Ae. albopictus* was tested by a four-arm olfactometer. The optimal compatibility and proportion of the odorants, Mix-5, was observed *via* orthogonal design analyses. The attractiveness of Mix-5 to *Ae. albopictus* in the laboratory was assessed using Mosq-ovitraps and Electric Mosquito Killers. In the field, the effectiveness of generic BG-Lure, Mix-5 and a control treatment was compared with a baited Biogents Sentinel trap (BGS-trap) using a Latin square design.

**Results:**

In the olfactometer experiments, the attractiveness of the selected candidate compounds at varying dilutions was poor when the individual compounds were used alone. The optimal combination, Mix-5, was generated based on orthogonal design analyses. In the laboratory, the average numbers of female *Ae. albopictus* mosquitoes attracted by the synthetic odorant blend Mix-5 were 27.00 and 27.50, compared with 12.00 and 14.83 for the control, when using Mosq-ovitraps and Electric Mosquito Killers, respectively. In the field, the average number of *Ae. albopictus* female mosquitoes trapped by Mix-5 was 9.67 females/trap, whereas the average numbers for BG-Lure and the control were 7.78 and 4.47, respectively. The lure also played an important role in attracting *Culex quinquefasciatus* mosquitoes, and the average numbers of *Cx. quinquefasciatus* female mosquitoes attracted by Mix-5, BG-Lure and the control were 18.78, 25.11 and 12.22, respectively.

**Conclusions:**

A human odor-based bait blend was developed and exhibited enhanced effectiveness at attracting *Ae. albopictus* This blend can be used to monitor and trap dengue vector mosquitoes in Chinese cities.

**Electronic supplementary material:**

The online version of this article (10.1186/s13071-019-3646-x) contains supplementary material, which is available to authorized users.

## Background

Vector-borne diseases pose a substantial and ever-increasing threat to public health and economies. The Asian tiger mosquito, *Aedes albopictus*, is a vector of several infectious diseases including dengue, chikungunya and Zika fevers, and is spreading throughout the world [1]. With no effective antiviral drugs or vaccines available for major *Aedes*-transmitted infectious diseases except yellow fever, the only viable method to prevent and control these diseases is control of the mosquito population. *Aedes albopictus* is a diurnally active species that exhibits relatively low sensitivity to light. The Biogents Sentinel (BGS) trap equipped with the standard BG-Lure (lactic acid, ammonia and hexanoic acid) [2, 3] and/or synergized with CO_2_ was initially designed for surveillance of the yellow fever mosquito, *Aedes aegypti* [3]. This tool has proven to be effective at trapping adult *Aedes* mosquitoes [4, 5]. However, mosquitoes use a variety of olfactory signals to locate the host, and the odor of human hosts is a mixture of many volatile compounds from the breath, skin, sweat and associated microbiota; therefore, BG-Lure may not be the most efficient attractant [6–8]. Furthermore, other studies have shown that in the absence of CO_2_, the BG-Lure cartridge alone cannot increase the capture of mosquitoes relative to an unbaited trap, indicating that CO_2_ is an essential addition for attracting *Ae. albopictus* [4, 9].

Host-seeking in mosquitoes is mediated primarily by olfaction. Many synthetic odor blends have been developed to survey and control mosquito vectors [10–12]. To enhance trapping efficiency, different chemoreceptors in receptor neurons, including odorant receptors (ORs), ionotropic receptors (IRs) and gustatory receptors (GRs), need to be activated. Intriguingly, cyclopentanone (C_5_H_8_O) activates the cpA CO_2_ receptor neuron on the maxillary palp of mosquitoes, suggesting that this molecule may be a potential CO_2_ substitute for mosquito surveillance [13]. A previous study found that cyclopentanone was less effective than CO_2_ as a mosquito attractant [14]. However, to our knowledge, no study has been conducted to assess whether the attractiveness of a blend can be enhanced with the incorporation of cyclopentanone.

A number of studies have been conducted to test the response of female mosquitoes to different odors in the laboratory and in field settings. Among the odors tested, 3-methyl-1-butanol, 6-methyl-5-hepten-2-one, hexanoic acid, 1-octen-3-ol, lactic acid and ammonia have been shown to attract *Aedes* female mosquitoes with fresh and/or incubated sweat or with incubated sweat liquid [2, 6, 8, 13, 15–20]. The most attractive blend or optimal combination of attractants at different concentrations to *Ae. albopictus* remains to be identified.

In this study, we selected seven odors that interacted with different classes of chemoreceptors: lactic acid, ammonia and hexanoic acid activate IRs in *Drosophila*, and many of these IRs are highly conserved in insects [21–23], while 3-methyl-1-butanol, cyclopentanone, 6-methyl-5-hepten-2-one (sulcatone) and 1-octen-3-ol activate ORs [24–26]. Additionally, cyclopentanone activates the cpA CO_2_ receptor neuron, which, in *Aedes* mosquitoes, harbors gustatory receptor 3 (GR3) [27]. The aim is to develop an attractive odor blend with enhanced attractiveness to improve the surveillance efficacy for *Ae.* albopictus mosquitoes.

## Methods

### Mosquitoes

The *Ae. albopictus* strain was obtained from the Center for Disease Prevention and Control (CDC), Guangdong Province, China. Mosquitoes were maintained under controlled conditions with a light:dark ratio of 14:10 h at a mean temperature of 27 ± 1 °C and a mean relative humidity (RH) of 75 ± 5%. The larvae were fed with fish food, and the adults were maintained on a 10% sugar solution. The mosquitoes had no prior access to a blood meal but had the opportunity to mate before the experiments.

### Compounds used to produce odorant blends

Lactic acid, hexanoic acid, 3-methyl-1-butanol, cyclopentanone, 1-octen-3-ol and 6-methyl-5-hepten-2-one were purchased from Sigma-Aldrich (St. Louis, MO, USA). Ammonia was purchased from Merck (Billerica, MA, USA). The purities and sources of the chemicals used in this study are listed in Additional file 1: Table S1. Details regarding the molecular structure and activated receptor of each odorant are shown in Additional file 2: Table S2.

### Olfactometer bioassays

**Fig. 1:**
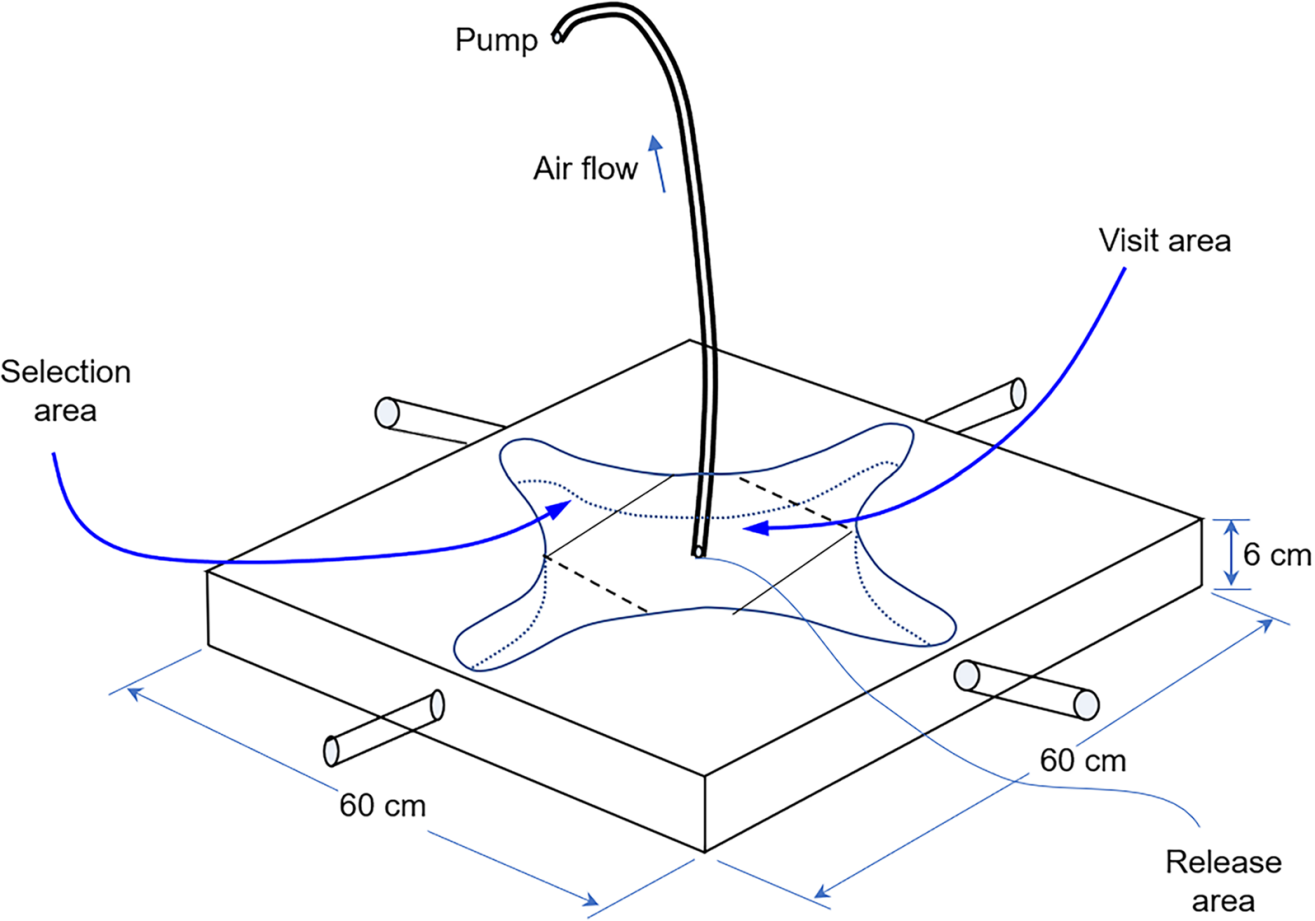
Pattern diagram of four-arm olfactometer (modified from Pettersson [30])

Y-tube, 3-port and 4-port olfactometers are available for similar experiments [28–30]. The Y-tube olfactometer design is good for testing one compound against a control [28], while the 3-port design is good for testing two compounds against a control [29]. Since we planned to test at least 3 concentrations of the same compound against a control, we used a modified version of the olfactometer originally described by Pettersson [30]. Briefly, the four-arm arena olfactometer (Shanghai Yuming Instrument Co., Shanghai, China) consisted of a large Perspex box (60 cm × 60 cm × 60 mm) connected to 4 inlet ports and 1 outlet port (Fig. 1). This olfactometer was composed of a star-shaped arena consisting of four regions and a neutral central zone that served as a mosquito holding space. The odor sources were contained in the glass arms, which were connected to holes on the sides of the olfactometer corresponding to the four regions within the olfactometer (Fig. 1). In this study, one arm served as the control, and the remaining arms were used as treatments. Mosquitoes were released in the middle (Fig. 1). If a mosquito remained in the central area (visit area in Fig. 1) during the experiments, the mosquito was considered to not be attracted by any odor. In contrast, if a mosquito moved and stayed in any of the ‘selection’ areas, the mosquito was considered to be attracted by an odor.

We placed 100 μl of the test stimulus on a 1-cm^2^ piece of filter paper, allowed 30 s for the solvent to evaporate, and then placed the paper in a treatment arm. For the control arms, 100 μl of hexane or water was applied to the filter paper. For each trial, 50 non-blood-fed 5–7-day-old female mosquitoes were used. Mosquitoes were released 30 min before odor stimulus was added, and the vacuum was initiated; the experiment was performed for approximately 15 min. The air flow rate was approximately 900 ml/min. The number of mosquitoes was counted in each selection area, and the corresponding odor was recorded. This process was repeated 6–10 times for each of the concentration.

### Screening of the most attractive odorant blends

The experiments were carried out under an ambient temperature of 26–28 °C and humidity of 65–85% in a 6 × 4 × 3 m laboratory. Approximately 50 female mosquitoes at 3–8 days post-eclosion and starved for 12 h were used for each test. The odor mixture was dispersed on 2-cm-diameter circular filter papers, which were placed in Mosq-ovitraps in the two diagonals of a Mongolian yurt (2.0 × 1.8 m) [31]. Each experiment consisted of an experimental group and a control group; the experiments were started at 17:00 h and sustained for 24 h. According to the olfactometer results and the composition of artificial sweat (EN 1811:1998): ammonia at 1% and lactic acid at 0.1% were used. Another five optimal combinations of odors at different concentrations was determined using an orthogonal design, i.e. each concentration was repeated 4 times (Additional file 3: Table S3). To increase the power of the experiments, the whole orthogonal experiment was repeated twice rather than once, i.e. each concentration was repeated 8 times. Mosq-ovitraps were cleaned with 30% methanol solution before being reused.

### Analysis of the effect of the most attractive odorant blends

#### Laboratory experiments

We tested our optimized combination in the Mongolian yurt using Mosq-ovitraps and Electric Mosquito Killer provided by Shunde Douhe Electronic Technology Co., Ltd. (Guangdong China). This process was repeated 6 times for each trap.

#### Field study

The attractiveness of the optimal blend obtained from the orthogonal experiment was further evaluated in the field against other currently used blends. Field studies were carried out from May 2016 to November 2017 on the campus of Southern Medical University (23°19′0″N, 113°34′0″E, 31 m above sea level), Guangzhou, China. Three study sites were chosen to represent a residential area, a park and a parterre. All the sites were examined for potential mosquito larval habitats before the experiments were conducted. We used a Latin square experimental design of days × sites × lures. A BG-Sentinel 2 trap (Biogents AG, Regensburg, Germany) was used as the “gold standard” trap [32, 33] for evaluation of the effectiveness of different attractants against *Aedes* mosquitoes.

We tested three types of attractants: (i) the BG-Lure, which consists of lactic acid, ammonia and caproic acid (hexanoic acid) [3]; (ii) the optimal blend obtained from the laboratory tests described above, which was dispersed by soaking nylon fabric as described by Okumu et al. [34]; and (iii) a blank control. The nylon strips were soaked in 1 ml of each chemical constituent at the optimal concentration for each attractant. The traps were placed at least 50 m apart and cleaned with 30% methanol solution before being reused. The Latin square design experiments were replicated three times. Mosquitoes collected from the traps were frozen and identified morphologically under a stereomicroscope using taxonomy keys [35].

### Statistical analysis

Differences among the mosquitoes captured in different arms of the olfactometer were compared using one-way ANOVA. In the Mongolian yurt experiments, data were analyzed using one-way ANOVA to screen the most attractive odorant blends, while a t-test was used to compare the optimized combination with control treatment in different type of trap. In the field study, the effects of the three types of attractant were tested while controlling for the variability among the three different sites (in this case, the Latin square number) and the three different trapping periods. The GLM procedure was performed using the number of female *Ae. albopictus* adults and female *Culex quinquefasciatus* mosquitoes collected in each trap as dependent variables and the attractants (BG-Lure, control and the optimal Mix-5) and the sites (of the trap) as fixed independent variables, and test dates were used as covariables. Multiple comparison procedures (Tukey’s HSD tests) were also performed to test significant differences in the number of mosquitoes caught among different treatments. Statistical analysis was performed using SPSS v.20.0 statistical software (IBM, Armonk, NY, USA). Prism v.6 (GraphPad Software, Inc., San Diego, USA) was used to plot the figures.

## Results

### Odor stimuli in four-arm olfactometer experiments

**Fig. 2:**
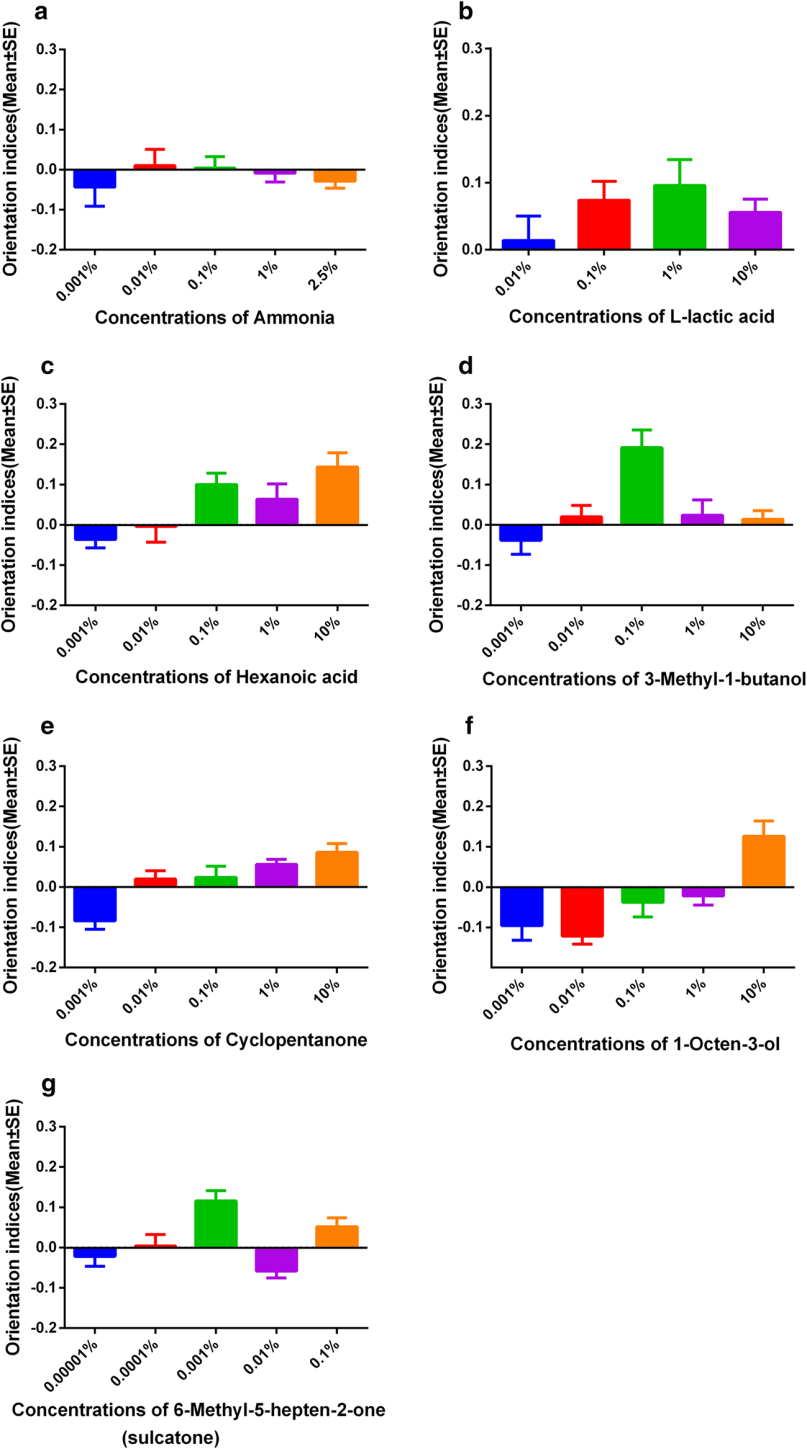
Comparison of the attractancies exhibited by different concentrations of each compound against female *Aedes albopictus* mosquitoes. **a** Ammonia. **b** l-Lactic acid. **c** Hexanoic acid. **d** 3-Methyl-1-butanol. **e** Cyclopentanone. **f** 1-Octen-3-ol. **g** 6-Methyl-5-hepten-2-one. Orientation index = (Nt − Nc)/T, where Nt is the number of mosquitoes trapped in the treatment chamber, Nc is the number of mosquitoes trapped in the control chamber and T is the total number of test mosquitoes. Bars represent the means ± SE (*n* = 6–10)

Among the 7 candidate odors tested, there were no significant differences among the five different concentrations of ammonia and four different concentrations of L-lactic acid (Fig. 2, Table 1). Hexanoic acid at 10% (*F*_(4, 45)_ = 5.03, *P* = 0.002), 3-methyl-1-butanol at 0.1% (*F*_(4, 45)_ = 6.52, *P* = 0.0003), 1-octen-3-ol at 10% (*F*_(4, 45)_ = 9.46, *P* < 0.0001) and sulcatone at 0.1% and 0.001% (*F*_(4, 45)_ = 8.06, *P* < 0.0001) attracted significantly higher numbers of mosquitoes than the control treatment and other concentrations of the same compound. For cyclopentanone in the range of 0.01% to 10% (*F*_(4, 45)_ = 9.15, *P* < 0.0001), the attractiveness increased with an increase in concentration (Fig. 2, Table 1).

**Table 1:** Flight orientation of female *Aedes albopictus* responses to various concentrations of selected compounds

Tested odorant	Concentration tested (%)	Orientation indices (mean ± SE)	*n*
l-Lactic acid	10	0.06 ± 0.02^a^	10
	1	0.10 ± 0.04^a^	10
	0.10	0.07 ± 0.03^a^	10
	0.01	0.01 ± 0.04^a^	10
Ammonia solution (NH_3_·H_2_O)	2.50	− 0.01 ± 0.02^a^	6
	1	− 0.01 ± 0.02^a^	6
	0.1	0.01 ± 0.03^a^	6
	0.01	0.01 ± 0.04^a^	6
	0.001	− 0.04 ± 0.05^a^	6
Hexanoic acid	10	0.14 ± 0.04^ab^	10
	1	0.06 ± 0.04^abcd^	10
	0.1	0.10 ± 0.03^abc^	10
	0.01	− 0.00 ± 0.04^bcd^	10
	0.001	− 0.04 ± 0.02^cd^	10
3-Methyl-1-butanol	10	0.01 ± 0.02^a^	10
	1	0.02 ± 0.04^a^	10
	0.1	0.19 ± 0.04^b^	10
	0.01	0.02 ± 0.03^a^	10
	0.001	− 0.04 ± 0.04^a^	10
Cyclopentanone	10	0.09 ± 0.02^a^	10
	1	0.06 ± 0.01^a^	10
	0.1	0.02 ± 0.03^a^	10
	0.01	0.02 ± 0.02^a^	10
	0.001	− 0.08 ± 0.02^b^	10
1-octen-3-ol	10	0.13 ± 0.04^a^	10
	1	− 0.02 ± 0.02^b^	10
	0.1	− 0.04 ± 0.04^b^	10
	0.01	0.12 ± 0.02^b^	10
	0.001	− 0.10 ± 0.04^b^	10
Sulcatone	0.1	0.05 ± 0.02^acde^	10
	0.01	− 0.06 ± 0.02^bde^	10
	0.001	0.12 ± 0.03^ac^	10
	0.0001	0.00 ± 0.03^abde^	10
	0.00001	− 0.02 ± 0.02^abde^	10

*Note*: Means for different concentration for the same compound indicated with same superscript letter represent no significant difference (Tukey’s HSD *post-hoc* test following a one-way ANOVA)

### Determination of the optimal combination

**Fig. 3:**
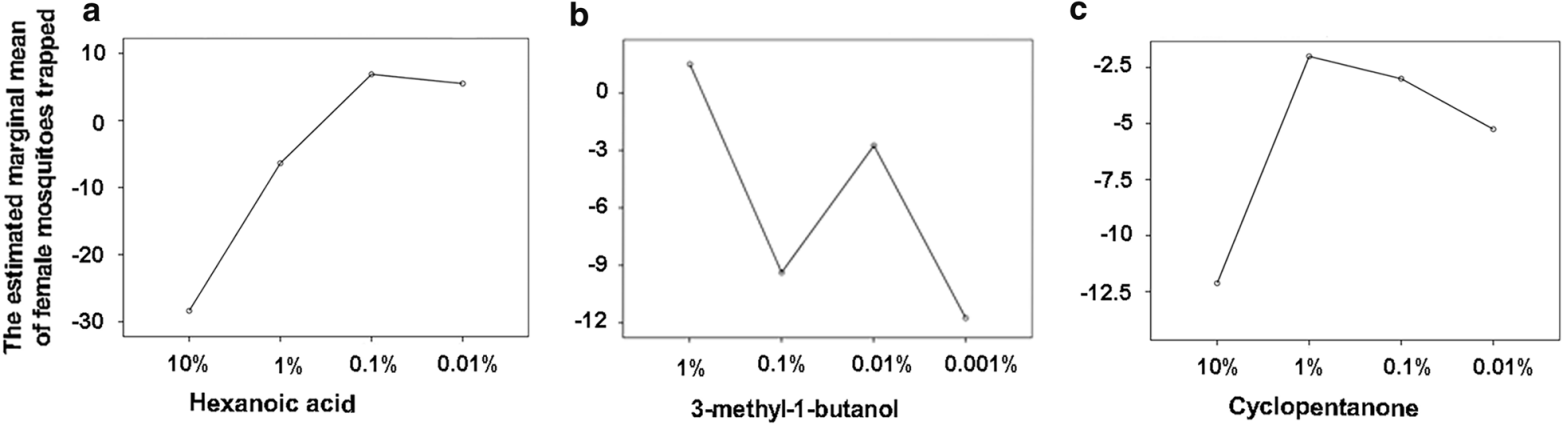
The estimated marginal means of compounds in Mix-5. **a** Hexanoic acid. **b** 3-Methyl-1-butanol. **c** Cyclopentanone

A mixture of hexanoic acid at 0.1%, 3-methyl-1-butanol at 1% and cyclopentanone at 1% (Table 2, Fig. 3) attracted the highest number of mosquitoes. The blend termed Mix-5, which contained ammonia (1%), lactic acid (0.1%), hexanoic acid (0.1%), 3-methyl-1-butanol (1%) and cyclopentanone (1%), was the most potent synthetic attractant for *Ae. albopictus*. As the addition of 1-octen-3-ol and sulcatone to Mix-5 did not significantly increase mosquito catches (Table 2, *P *> 0.05), these compounds were excluded from the optimal mixture (Additional file 4: Figure S1).

**Table 2:** The analysis of individual odor effect (*R*^2^ = 0.914, adjusted *R*^2^ = 0.833)

Source	Type III sum of squares	*df*	Mean square	*F*-value	*P*-value
Corrected model	8043.22	15	536.22	11.34	< 0.0001
Intercept	1001.28	1	1001.28	21.18	< 0.0001
Hexanoic acid	6385.09	3	2128.37	45.02	< 0.0001
3-methyl-1-butanol	884.84	3	294.95	6.24	0.005
1-octen-3-ol	155.59	3	51.87	1.10	0.379
Sulcatone	118.34	3	39.45	.83	0.494
Cyclopentanone	499.34	3	166.45	3.52	0.039
Error	756.50	16	47.28		
Total	9801.00	32			
Corrected total	8799.72	31			

*Abbreviation*: df, degrees of freedom

### Effect of Mix-5 on mosquito catches in two types of mosquito traps

**Fig. 4:**
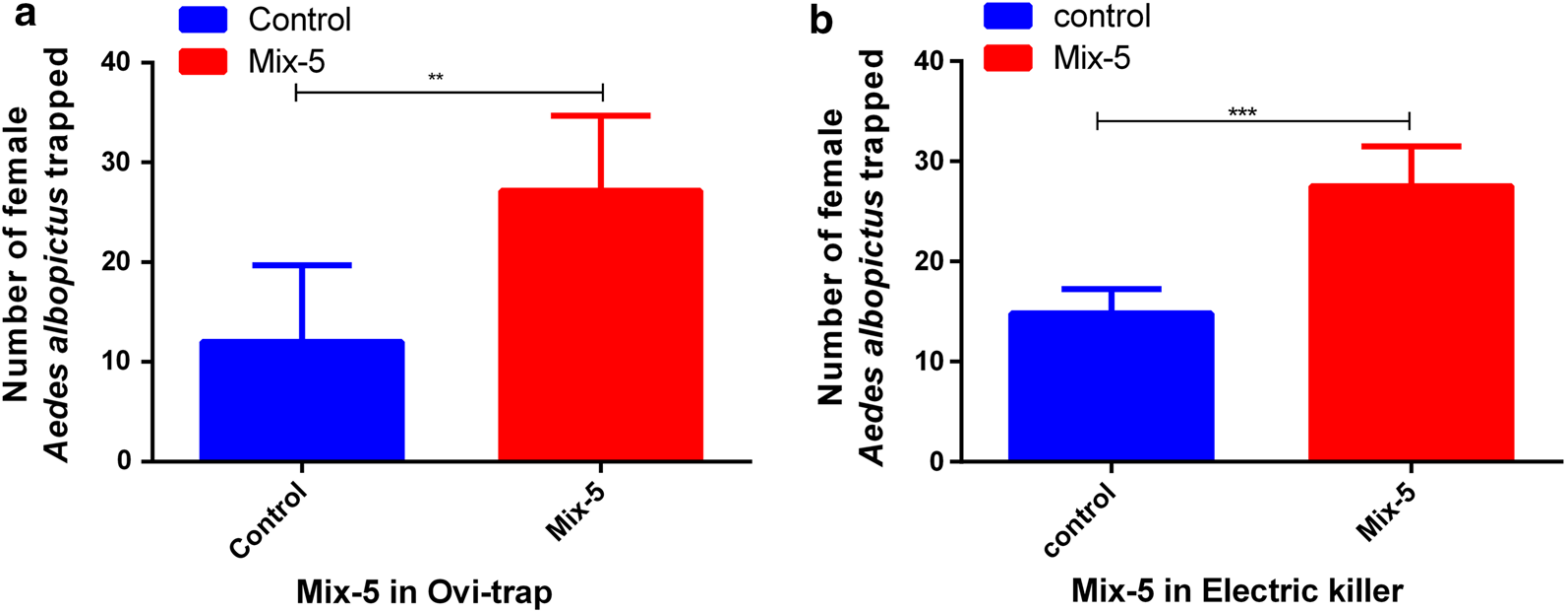
Comparison of attractancy against female *Aedes albopictus* mosquitoes. **a** Mean catches by Mosq-ovitrap. **b** Mean catches by electric mosquito killer. Bars represent the means ± SE (*n* = 6). *P < 0.05, **P < 0.01, ***P < 0.001

The average number of mosquitoes attracted by Mix-5 with Mosq-ovitraps was 27.00 ± 3.07, compared to 12.00 ± 3.14 for the control (*t*_(10)_ = 3.45, *P* = 0.006) (Fig. 4a).

The average number of mosquitoes attracted by Mix-5 was 27.50 ± 1.63 with the use of Electric Mosquito Killers, compared to 14.83 ± 1.00 for the control (*t*_(10)_ = 6.67, *P* < 0.0001) (Fig. 4b).

### Field study

**Fig. 5:**
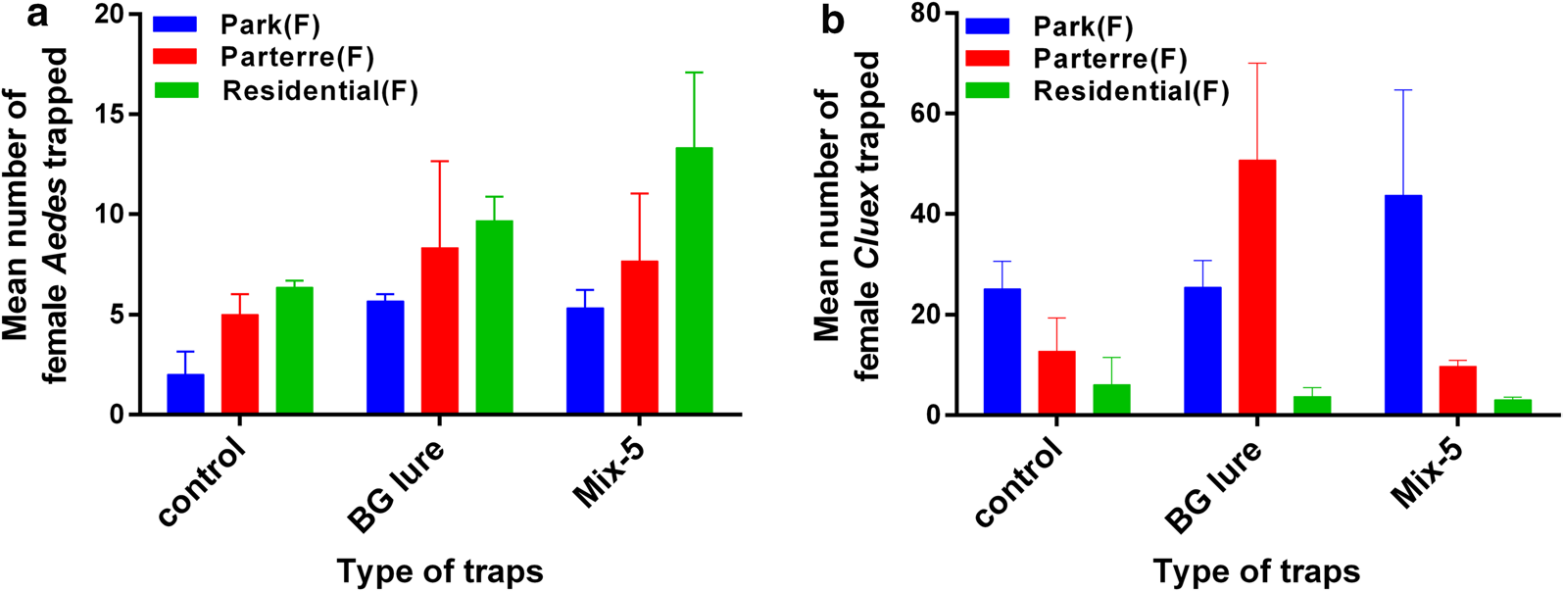
Female mosquitoes collected in the traps baited with three different attractants in the field. **a** *Aedes albopictus*. **b** *Culex quinquefasciatus*. Bars represent the means ± SE (*n* = 9)

A total of 1595 adult mosquitoes were collected during the study period. They consisted of 358 *Ae. albopictus* (197 females and 161 males), 1234 *Cx. quinquefasciatus* (505 females and 729 males) and 3 female *Armigeres subalbtus* mosquitoes. In total, 63 (40 females and 23 males, 17.60%), 164 (70 females and 94 males, 45.80%) and 131 (87 females and 44 males, 36.60%) *Ae. albopictus* mosquitoes were captured by traps baited with the control, BG-Lure and Mix-5, respectively (Table 3). The number of *Ae. albopictus* mosquitoes caught varied significantly among the three types of lures (Fig. 5a, GLM; *F*_(2, 27)_ = 5.49, *P* = 0.017 for females). In addition, the number of female *Ae. albopictus* mosquitoes varied significantly at different locations (GLM; *F*_(2, 27)_ = 4.33, *P* = 0.03 for females), but there was no difference among the days of trap deployment (GLM; *F*_(8, 27)_ = 1.95, *P* = 0.132 for females).

**Table 3:** Mosquitoes collected in the traps baited with three different attractants in the field

Bait	Species	Sex	Total	Mean ± SE	*n*
Control	*Aedes albopictus*	Female	40	4.44 ± 1.13^a^	9
		Male	23	2.56 ± 1.67^a^	9
	*Culex quinquefasciatus*	Female	110	12.22 ± 6.75^b^	9
		Male	208	23.11 ± 6.50^b^	9
Mix-5	*Aedes albopictus*	Female	87	9.67 ± 1.13^b^	9
		Male	44	4.89 ± 1.67^a^	9
	*Culex quinquefasciatus*	Female	169	18.78 ± 4.0^b^	9
		Male	158	17.56 ± 6.50^b^	9
BG-lure	*Aedes albopictus*	Female	70	7.78 ± 1.13^a^	9
		Male	94	10.44 ± 1.67^b^	9
	*Culex quinquefasciatus*	Female	226	25.11 ± 4.0^b^	9
		Male	363	40.33 ± 6.50^b^	9

*Note*: The same superscript letter indicates a non-significant difference

For *Cx. quinquefasciatus*, 318 (110 females and 208 males, 25.77%), 589 (226 females and 363 males, 47.73%) and 327 (169 females and 158 males, 26.50%) mosquitoes were captured in the control, BG-Lure and Mix-5 traps, respectively. Table 3 shows the numbers of mosquitoes collected in the traps baited with the three different attractants in the field. The number of *Cx. quinquefasciatus* mosquitoes caught was not significantly different among the three types of lures (Fig. 5b, GLM; *F*_(2, 27)_ = 2.61, *P* = 0.109 for females) but was significantly different among the different locations (GLM; *F*_(2, 27)_ = 10.03, *P* = 0.002 for females) and different days (GLM; *F*_(8, 27)_ = 6.42, *P* = 0.001 for females)

## Discussion

Natural olfactory stimuli are typically mixtures of chemical constituents, and the identities, concentrations and ratios of these constituents are important for many odor-mediated behaviors. Different odors exhibit varying abilities to elicit behavioral responses. Our research focused on developing a synthetic odor blend to improve the efficacy of trapping *Ae. albopictus*.

Ammonia, lactic acid and carboxylic acid synergistically affect the host-seeking behavior of *Anopheles gambiae* (*sensu stricto*) [2]. Kröckel et al. [3] showed that lactic acid, ammonia and hexanoic acid at a fixed ratio attracts a large number of adult *Ae. aegypti* mosquitoes. A recent study demonstrated that hexanoic acid is a superior odor bait for *Ae. aegypti* compared to the commercially available BG-Lure [36]. Our results indicated that among the five concentrations of hexanoic acid tested, 10% was the most effective for attracting *Ae. albopictus*. In our study, cyclopentanone at high dosages (1% or 10%) attracted large numbers of mosquitoes. This result is in agreement with findings reported by Tauxe et al. [13] that cyclopentanone at a 20% concentration collected more *Cx. quinquefasciatus* mosquitoes than CO_2_-baited traps. Notably, 3-methyl-1-butanol at 0.001% significantly facilitated the trapping of *Anopheles* mosquitoes in the field [37]. Our results showed that female *Ae. albopictus* mosquitoes are attracted by 0.1% 3-methyl-1-butanol in the laboratory. We speculate that different mosquito species or experimental conditions may account for this result, which warrants further investigation.

In one previous study, 6-methyl-5-hepten-2-one was found to be a weak attractant to *Ae. aegypti* [38]. In contrast, another study suggested that inhibitory effects occur at low concentrations of 6-methyl-5-hepten-2-one [39]. Our study found that 6-methyl-5-hepten-2-one attracted mosquitoes at concentrations of 0.001% and 0.1% but reduced attractiveness at other concentrations. These results suggest: (i) mixtures of odorants exhibited improved attractiveness to mosquitoes relative to single odorants; and (ii) the odor mixtures work in a manner that is largely dependent on both the proportion and dilution of the ingredient. Our findings are consistent with the synergism and combinatorial coding for mixture perception reported for *Drosophila* [40].

In the field experiments, our results were consistent with previous investigations that showed BG-Sentinel traps were efficient tools for monitoring both *Aedes* and *Culex* mosquitoes [41]. Notably, the field evaluations demonstrated that Mix-5 was more attractive than BG-Lure to *Ae. albopictus* females, but not *Cx quinquefasciatus* female mosquitoes. The use of Mix-5 in combination with BG-Sentinel or CDC traps can potentially improve the efficacy of *Ae. albopictus* and *Cx. quinquefasciatus* mosquito surveillance. Moreover, when using Mix-5, specialized equipment is not required to carry the chemicals, thereby avoiding traditional methods for CO_2_ delivery, thus saving material cost and labor. Notably, other cues such as moisture, heat and visual cues also affect the recognition of hosts by mosquitoes [27, 42], and different habitats may be suitable for different mosquito species, which may partially explain why different types of sites had different effects. Different dispensing methods should also be tested in future studies.

It should be noted that BG-Lure, although not the most efficient bait, is widely used with consistent results across multiple settings. In this study, we found that the Mix-5 lure was significantly more efficient in attracting *Ae. albopictus* relative to control treatment in the laboratory setting, and Mix-5 was slightly more efficient than BG-Lure in the field setting. However, whether Mix-5 is consistently more efficient than BG-Lure in different settings needs to be further investigated. Furthermore, BG-Lure is good for attracting different species of mosquitoes, for example *Ae. aegypti*, *Ae. albopictus* and *Culex pipiens* mosquitoes, but whether Mix-5 is a good lure for *Ae. aegypti* and *Culex pipiens* mosquitoes would be a subject for further investigation.

## Conclusions

We developed and evaluated a human odor-based attractant blend that enhanced the effectiveness of attracting adult *Ae. albopictus* mosquitoes. Exploiting the behavioral responses of different types of mosquito chemoreceptors to host stimuli provides new paradigms for the development of new surveillance and control tools for major disease vectors.

## Additional files

**Additional file 1: Table S1.** List of odorants used in the experiments against *Ae. albopictus* females.

**Additional file 2: Table S2.** The molecular structure and activated receptor of each odorant.

**Additional file 3: Table S3.** The orthogonal design list.

**Additional file 4: Figure S1.** The estimated marginal means of **a** 1-octen-3-ol and **b** 6-methyl-5-hepten-2-one.

## Data Availability

The datasets supporting the conclusions of this article are included within the article and its additional files.

## Abbreviations

BGS: Biogents Sentinel trap
ORs: odorant receptors
IRs: ionotropic receptors
GRs: gustatory receptors
GR3: gustatory receptor 3
OR8: odorant receptor8
OR4: odorant receptor4
C_5_H_8_O: cyclopentanone
cpA: CO_2_-responsive neurons
