# Supplementary material for: Enhancing attraction of the vector mosquito Aedes albopictus by using a novel synthetic odorant blend
Source: Parasit Vectors. 2019 Jul 30;12:382. doi: 10.1186/s13071-019-3646-x (PMC6668062; doi:10.1186/s13071-019-3646-x)
Supplement: Supplementary file 1 — Additional file 1: Table S1. List of odorants used in the experiments against Ae. albopictus females. [file 13071_2019_3646_MOESM1_ESM.docx]

**Additional file 1: Table S1. List of odorants used in the experiments against *Aedes albopictus* females**

| **CAS number** | **Odorant** | **Purity (%)** | **Molecular weight (g/mol)** | **Source** |
| --- | --- | --- | --- | --- |
| 50-21-5 | L-lactic acid | ≥85% | 90.08 | Sigma-Aldrich |
| 1336-21-6 | Ammonia solution | ≥25% | 35.05 | Merck |
| 142-62-1 | Hexanoic acid | ≥98% | 116.16 | Sigma-Aldrich |
| 120-92-3 | Cyclopentanone | ≥99% | 84.12 | Sigma-Aldrich |
| 110-93-0 | Sulcatone | ≥98% | 126.2 | Sigma-Aldrich |
| 3391-86-4 | 1-Octen-3-ol | ≥98% | 128.21 | Sigma-Aldrich |
| 123-51-3 | 3-Methyl-1-butanol | ≥98.5% | 88.15 | Sigma-Aldrich |
