# Supplementary material for: Enhancing attraction of the vector mosquito Aedes albopictus by using a novel synthetic odorant blend
Source: Parasit Vectors. 2019 Jul 30;12:382. doi: 10.1186/s13071-019-3646-x (PMC6668062; doi:10.1186/s13071-019-3646-x)
Supplement: Supplementary file 2 — Additional file 2: Table S2. The molecular structure and activated receptor of each odorant. [file 13071_2019_3646_MOESM2_ESM.docx]

| **Additional file 2: Table S2. The molecular structure and their activated receptor of each odorant** | | | | |
| --- | --- | --- | --- | --- |
|  |  |  |  |  |
| **Tested odorants** | | **Receptors** | **Concentrations  tested** | **Reference** |
|  |  |  |  |  |
| L-lactic acid 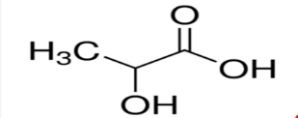 | IRs | | 10% | [2-3][6-8][19][21] |
|  |  |  | 1% |  |
|  |  |  | 0.1% |  |
|  |  |  | 0.01% |  |
| Ammonia solution （NH_3_•H_2_O） | | IRs | 2.5% | [2-3] |
|  |  |  | 1% |  |
|  |  |  | 0.1% |  |
|  |  |  | 0.01% |  |
|  |  |  | 0.001% |  |
| Hexanoic acid 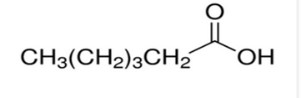 | | IRs | 10% | [23][17] |
|  |  |  | 1% |  |
|  |  |  | 0.1% |  |
|  |  |  | 0.01% |  |
|  |  |  | 0.001% |  |
| 3-Methyl-1-butanol 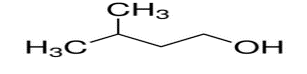 | | ORs | 10% | [9] |
|  |  |  | 1% |  |
|  |  |  | 0.1% |  |
|  |  |  | 0.01% |  |
|  |  |  | 0.001% |  |
| Cyclopentanone 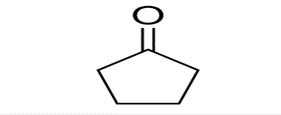 | | GR3 | 10% | [13-14] |
|  |  |  | 1% |  |
|  |  |  | 0.1% |  |
|  |  |  | 0.01% |  |
|  |  |  | 0.001% |  |
| 1-octen-3-ol 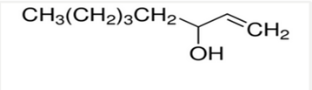 | | OR8 | 10% | [16][18] |
|  |  |  | 1% |  |
|  |  |  | 0.1% |  |
|  |  |  | 0.01% |  |
|  |  |  | 0.001% |  |
| Sulcatone 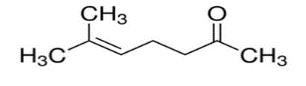 | | OR4 | 0.1% | [24] |
|  |  |  | 0.01% |  |
|  |  |  | 0.001% |  |
|  |  |  | 0.0001% |  |
|  |  |  | 0.00001% |  |
| *Abbreviations:* ORs (odorant receptors), IRs (ionotropic receptors), GRs (gustatory receptors) GR3: gustatory receptor 3, OR8: odorant receptor8, OR4: odorant receptor4. | | | | |
