# Supplementary material for: Enhancing attraction of the vector mosquito Aedes albopictus by using a novel synthetic odorant blend
Source: Parasit Vectors. 2019 Jul 30;12:382. doi: 10.1186/s13071-019-3646-x (PMC6668062; doi:10.1186/s13071-019-3646-x)
Supplement: Supplementary file 3 — Additional file 3: Table S3. The orthogonal design list. [file 13071_2019_3646_MOESM3_ESM.docx]

**Additional file 3: Table S3. The Orthogonal design list**

| **Number** | **Hexanoic acid**  **(4)** | **3-Methyl-1-butanol**  **(4)** | **1-octen-3-ol**  **(4)** | **Sulcatone**  **(4)** | **Cyclopenta-none**  **(4)** |
| --- | --- | --- | --- | --- | --- |
| 1 | 1% | 0.1% | 10% | 0.01% | 1% |
| 2 | 0.1% | 0.01% | 10% | 0.001% | 0.1% |
| 3 | 0.1% | 0.1% | 1% | 0.1% | 0.01% |
| 4 | 1% | 1% | 0.1% | 0.001% | 0.01% |
| 5 | 1% | 0.01% | 1% | 0.0001% | 10% |
| 6 | 0.1% | 0.001% | 0.1% | 0.01% | 10% |
| 7 | 0.1% | 1% | 0.01% | 0.0001% | 1% |
| 8 | 10% | 0.1% | 0.1% | 0.0001% | 0.1% |
| 9 | 10% | 0.001% | 1% | 0.001% | 1% |
| 10 | 0.01% | 1% | 1% | 0.01% | 0.1% |
| 11 | 0.01% | 0.01% | 0.1% | 0.1% | 1% |
| 12 | 0.01% | 0.001% | 10% | 0.0001% | 0.01% |
| 13 | 1% | 0.001% | 0.01% | 0.1% | 0.1% |
| 14 | 10% | 0.01% | 0.01% | 0.01% | 0.01% |
| 15 | 10% | 1% | 10% | 0.1% | 10% |
| 16 | 0.01% | 0.1% | 0.01% | 0.001% | 10% |
