# Supplementary figures and images for: Enhancing attraction of the vector mosquito Aedes albopictus by using a novel synthetic odorant blend
Source: Parasit Vectors. 2019 Jul 30;12:382. doi: 10.1186/s13071-019-3646-x (PMC6668062; doi:10.1186/s13071-019-3646-x)

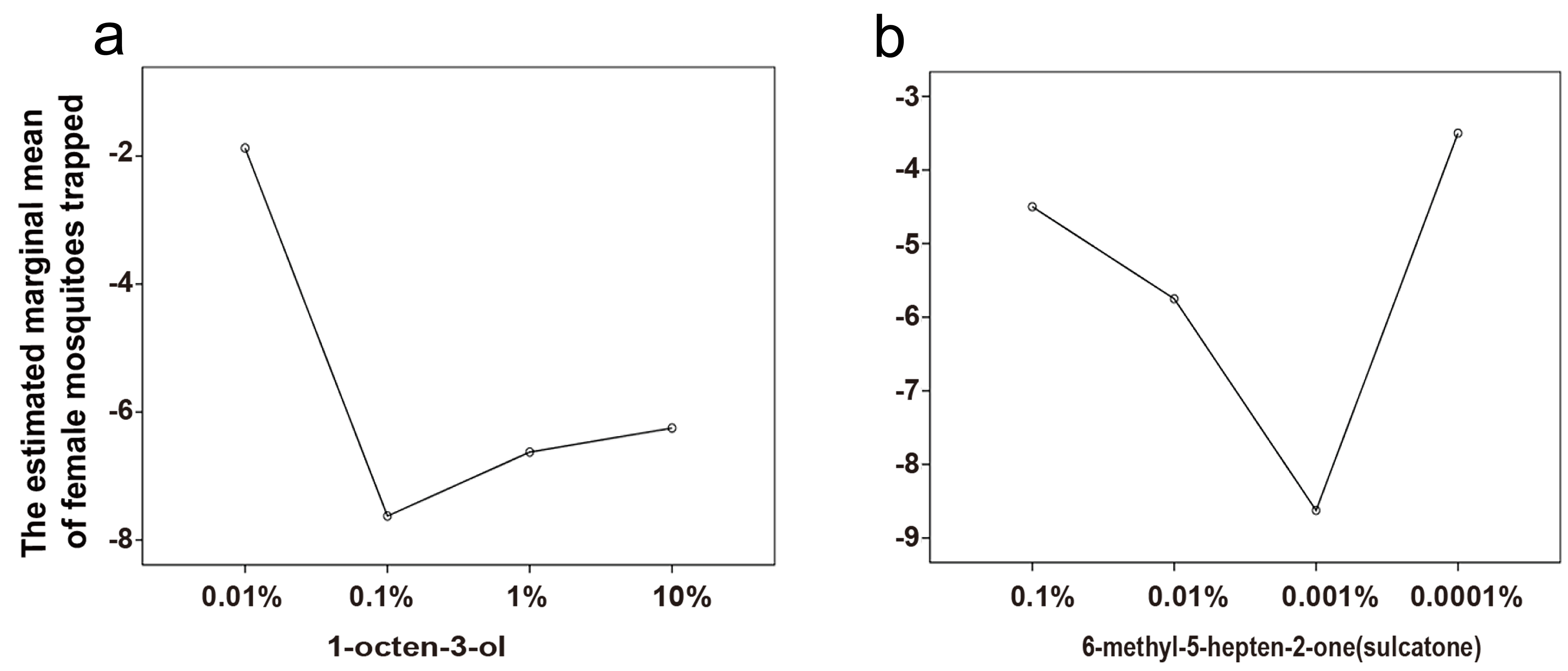

Supplement: Supplementary file 4 — Additional file 4: Figure S1. The estimated marginal means of a 1-octen-3-ol and b 6-methyl-5-hepten-2-one. [file 13071_2019_3646_MOESM4_ESM.tif]
